# Effect of Alternative Palatal Root Access Technique on Fracture Resistance of Root Canal Treated Maxillary Fourth Premolar Teeth in Dogs

**DOI:** 10.3389/fvets.2020.600145

**Published:** 2020-12-11

**Authors:** Jennifer Matelski, Aaron Rendahl, Stephanie Goldschmidt

**Affiliations:** Department of Veterinary Clinical Sciences, College of Veterinary Medicine, University of Minnesota, St. Paul, MN, United States

**Keywords:** tooth fracture, small animal dentistry, root canal, maxillary fourth premolar tooth, veterinary endodontics, fracture resistance

## Abstract

A biomechanical study was performed to identify the effect of different treatment methods for difficult to instrument palatal roots on the fracture resistance of root canal treated maxillary fourth premolar teeth in dogs. Forty maxillary fourth premolar teeth with surrounding alveolar bone were harvested from beagle cadavers. Inclusion criteria included maxillary fourth premolars with no evidence of disease and similar distal root canal volumes on radiographic evaluation. The teeth were randomly divided into a control group and three treatment groups based on the endodontic treatment technique for the palatal root. The control group had a single 2 mm transcoronal access on the mesiobuccal aspect of the tooth to allow instrumentation of both the mesiobuccal and palatal root through a single small access. Alternative treatment modalities that are described for difficult to instrument palatal roots investigated in this study included enlarging the transcoronal mesiobuccal access to 4 mm, making an additional access directly over the palatal root (2 mm), and hemisection with extraction of the palatal root. All teeth had the same distal root access size (2 mm) and relative location. After access, all teeth were filed, shaped, obturated, and restored in the same fashion. Axial compression testing was performed at an angle of 60 degrees to the long axis of the tooth using a universal materials testing machine. The maximum force prior to fracture was determined for each tooth based on a force vs. deflection curve. The mean maximum force prior to fracture for all teeth was 831 N. No significant difference in mean fracture resistance was identified between the control group and treatment groups or between the different treatment groups themselves. Thus, when faced with a difficult to instrument palatal root, the treatment method chosen should be based on operator preference and experience.

## Introduction

Tooth fracture in dogs is a common condition with a reported prevalence as high as 27% (1). Strategic teeth, which include the canine and carnassial teeth (maxillary fourth premolar and mandibular first molar) are the most commonly injured. Specifically, in a study evaluating the prevalence of dentoalveolar trauma in the canine population, 39% of all fractured teeth were premolars and 33.3% of all fractured teeth were canines (1). When evaluating the rate of injury of the carnassial teeth only, the maxillary fourth premolar is the most commonly fractured, accounting for 92.2% of all fractured carnassial teeth (1).

Fracture of the tooth often leads to pulp exposure, and accordingly, nearly half (49.6%) of reported dentoalveolar injuries are enamel-dentin-pulp fractures (1). When the pulp is exposed, it becomes overwhelmed by bacteria from the oral cavity, eventually resulting in pulp necrosis and secondary development of periapical periodontitis (2). Therefore, a tooth that has suffered an enamel-dentin-pulp fracture, also known as a complicated crown fracture, requires either surgical extraction or endodontic therapy (2).

Due to the strategic nature of the maxillary fourth premolar tooth, many owners elect endodontic therapy over extraction. While specific endodontic therapy depends on the age of the patient and timing of the injury, the most commonly performed endodontic treatment is standard root canal therapy (2). The goal of endodontic therapy is to maintain the function of the tooth within the oral cavity for the remainder of the patient's life. Maintenance of the tooth within the oral cavity relies on both the success of the endodontic procedure (successful prevention or healing of periapical infection) and continued fracture resistance of the tooth post endodontic therapy (2, 3).

The ability to resist further fracture after endodontic therapy is of particular concern for the carnassial teeth, as these teeth undergo the highest occlusal forces within the oral cavity during normal chewing behavior (4–6). A previous study measuring bite forces in dogs under anesthesia using electrodes to stimulate the masticatory muscles found a bite force range of 147–926 N for the canine teeth, and a range of 574–3,417 N for the carnassial teeth (5). An additional study utilizing finite element analysis in a dingo also found a significant difference in bite forces between the canine and carnassial teeth, with the calculated bite force range being nearly double for carnassial teeth (6). Given the high forces the maxillary fourth premolar tooth must sustain, a key principal of endodontic therapy for this tooth is preservation of strength and fracture resistance.

Previous studies in human dentistry have shown that endodontically treated teeth have lower fracture resistance than vital teeth (7–13). Many factors may contribute to this decreased strength including increased brittleness of dentin secondary to desiccation, removal of hard tissue during access and instrumentation, and lack of sensory feedback from the tooth (3, 14–20). However, it has been shown that the largest contributor to fracture resistance post endodontic therapy is access method, with more conservative access design conferring increased tooth strength (14–20). To the authors' knowledge, there are currently no studies in veterinary medicine evaluating the effects of endodontic therapy or specific access design on the fracture resistance of root canal treated teeth.

Compared to other single rooted, or even double rooted teeth, access design and endodontic treatment options are more varied for the maxillary fourth premolar. This tooth has three separate roots; two buccal roots, and one mesial palatal root. Due to its location, often, the most challenging aspect of root canal therapy in the maxillary fourth premolar tooth is complete instrumentation of the palatal root canal. The goal of instrumentation is to completely remove necrotic pulp material allowing for sterilization of the pulp cavity and shaping of the root canal to facilitate obturation (2). Thus, incomplete instrumentation can result in root canal failure (2).

**Figure 1:**
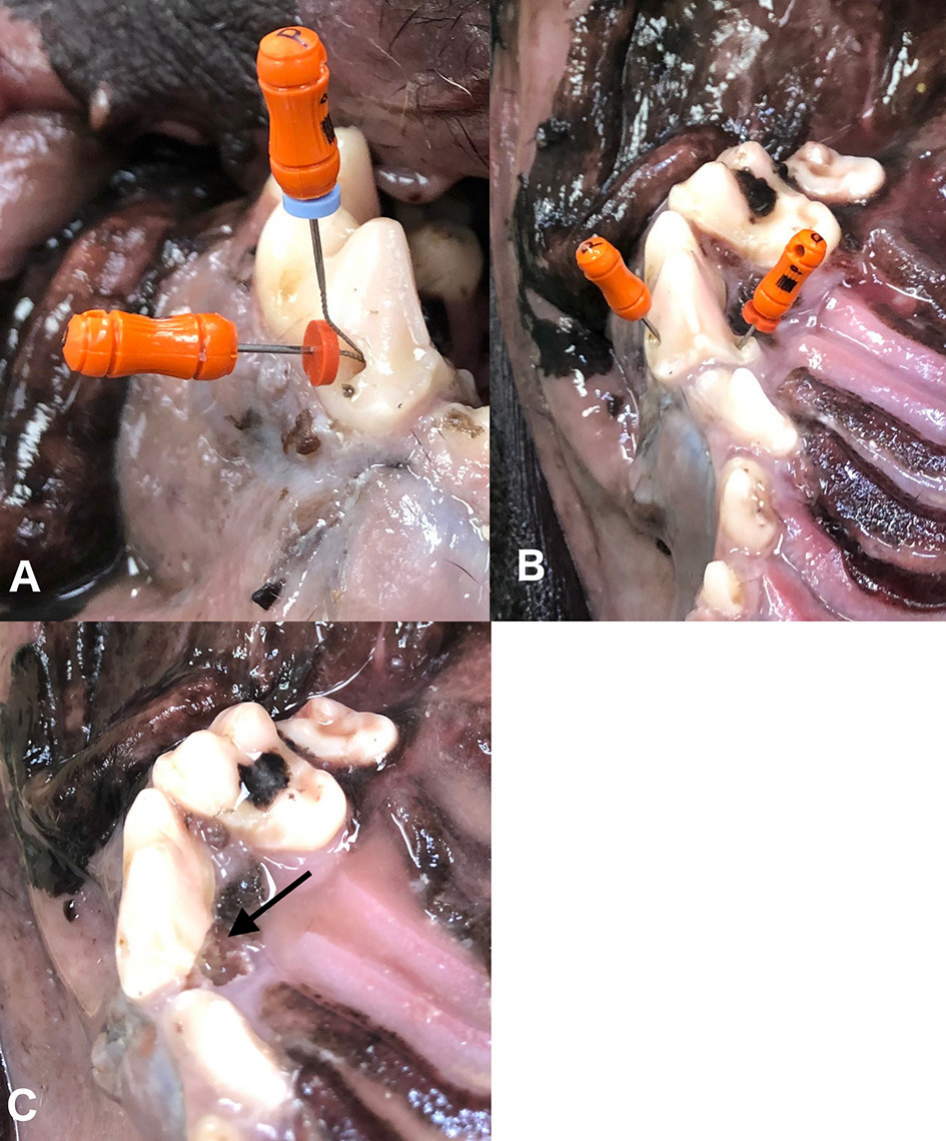
Palatal root endodontic treatment options depicted in an intact maxillary arch. Transcoronal mesiobuccal access to allow instrumentation of both the mesiobuccal and palatal roots through a single access **(A)**. Access directly over the palatal root and mesiobuccal root separately for instrumentation **(B)**. Hemisection and extraction of the palatal root with arrow pointing to area of hemisection and extraction **(C)**.

The most commonly recommended and performed access design to allow complete instrumentation of the palatal root canal is a transcoronal approach through a single mesial buccal access (2, 21) (Figure 1A). However, this access design is often not adequate to achieve complete instrumentation of the palatal pulp chamber, especially in older patients with narrower pulp cavities. In instances where it is difficult to enter and/or completely instrument the palatal root, there are three described alternative treatment options including increasing the size of the transcoronal buccal access (Figure 1A), adding an additional access directly over the palatal pulp chamber (Figure 1B), or performing palatal root hemisection with extraction (Figure 1C) (2, 22). However, the effects of these alternative palatal root treatment techniques on fracture resistance following standard root canal therapy is unknown. It was hypothesized that enlarging the mesiobuccal access would result in the greatest reduction in fracture resistance.

The aim of the present study was to identify the effect of alternative palatal root treatment techniques on fracture resistance of root canal treated maxillary fourth premolar teeth in dogs.

## Materials and Methods

### Specimen Collection and Screening

Thirty-one adult (~2-year-old) cadaver heads of beagle dogs previously euthanized for causes unrelated to this study were evaluated. Structurally intact maxillary fourth premolar teeth with no radiographic signs of periodontal disease, endodontic disease, or tooth resorption were included. Only teeth with subjectively similar pulp cavity widths on radiographic evaluation were included. In order to further standardize the maxillary fourth premolar teeth, the distal root canal volume of each tooth was calculated. Specifically, the length and width of the distal root canal were measured on a lateral radiograph using the measurement capabilities of the viewing software. The distal root canal volume was then calculated using the formula for a cone, *V* = 1/3(π*r*^2^ *h*), where r is the distal root canal width at the level of the cemento-enamel-junction and h is the distal root canal height from the apical end of the root canal to the level of the cemento-enamel-junction. A total of 30 teeth fell within ±25% of the mean distal root canal volume, while an additional 10 teeth fell within ±40% of the mean distal root canal volume. In order to evenly distribute teeth with greater distal root canal variation, random group assignments were performed in two steps. First the 30 teeth within 25% of the mean were randomly assigned to one of the four groups. After distribution of the more similar teeth, the 10 teeth within 40% of the mean were randomly assigned to one of the four groups, creating 4 groups with a total of 10 teeth each.

Cadaver heads were frozen until the time of tooth harvesting. The maxillary fourth premolar teeth and surrounding alveolar bone were collected as previously described (23). Briefly, after removing soft tissue, the maxillary fourth premolar teeth and surrounding alveolar bone were collected by performing osteotomies at the distal aspect of the third premolar, mesial aspect of the first molar, and dorsolateral and palatal aspects of the maxilla using a #701, cross-cut, fissure bur on a high speed dental handpiece.

After collection, all samples were photographed and radiographed to confirm teeth were not damaged during the collection process and were collected in their entirety. The crown height and crown diameter of each tooth was measured using a digital caliper. The crown height was measured from the furcation of the buccal roots to the most coronal point of the crown and the crown diameter was measured from the most mesial to most distal aspects of the crown as previously described (23). The teeth were then stored in lactated Ringer's solution at 15.8°F (−9°C) until the time of endodontic treatment.

### Endodontic Treatment

**Figure 2:**
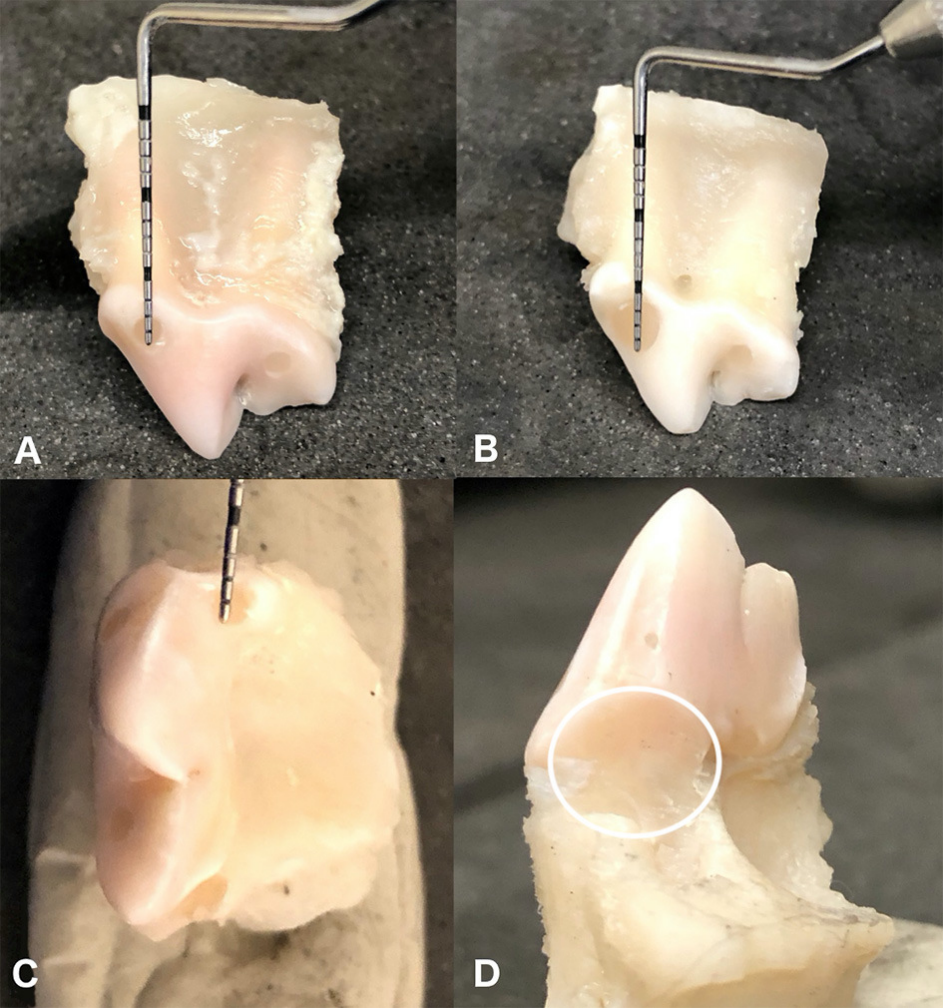
Appearance of en-bloc maxillary fourth premolar teeth following endodontic treatment for each different treatment group. Control group with a single transcoronal mesiobuccal access 2 mm in diameter **(A)**. Enlarged mesiobuccal access group with a mesiobuccal access 4 mm in diameter **(B)**. Direct palatal access group with an additional 2 mm access directly over the palatal root **(C)**. Hemisection group which had the palatal root hemisected and surgically extracted, circle outlining area of hemisection and extraction **(D)**. All groups had a single distal access 2 mm in diameter.

Prior to endodontic treatment, the maxillary fourth premolar teeth were removed from refrigeration and allowed to warm to room temperature (20°C). Specific endodontic treatment per each group is described below and depicted in Figures 1, 2.

**Figure 3:**
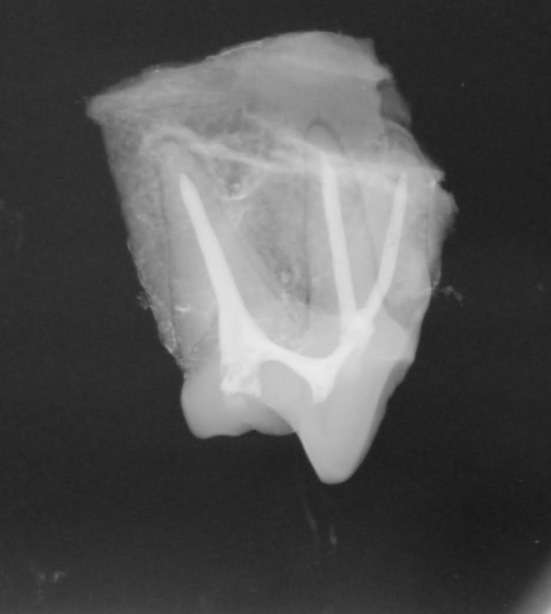
Radiographic example of acceptable root canal obturation.

In the control group (*n* = 10) the maxillary fourth premolars were treated with standard root canal therapy utilizing a 2 mm diameter transcoronal mesiobuccal access to allow instrumentation of the palatal and mesiobuccal roots, and a 2 mm distal root canal access (Figure 2A). This access size was chosen because, based on clinical experience, it visually represented a “conservative” access size that would still allow complete instrumentation of the root canal system. Access into the pulp chambers was made with a #330 pear bur on a high-speed dental hand piece. Access size was slowly enlarged in a circular motion until the diameter measured 2 mm with a periodontal probe in both a mesial to distal direction as well as a coronal to apical direction. Following access, the root canals were cleaned, filed, and shaped using standard root canal instrumentation and a step-back technique (2). Irrigation was performed throughout instrumentation using 5.25% sodium hypochlorite. The final irrigation cycle was performed using ethylenediaminetetraacetic acid (EDTA) for 1 min (24) followed by irrigation with 5.25% sodium hypochlorite and a final rinse with saline. Obturation was then performed using a resin-based sealer cement (AH Plus Jet Root Canal Sealer; Dentsply Sirona, Charlotte, NC) and a single master gutta percha cone (Gutta Percha Points, Diadent, Burnaby, BC). Obturation technique was chosen based on clinical preference. Coronal restoration was performed using a glass ionomer (Ionoseal, Voco, Cuhaven, Germany) intermediate layer followed by a final composite restoration (Aelite Flo, Bisco, Anaheim, CA). All teeth were radiographed after obturation, but prior to restoration, to ensure no significant voids in filling (Figure 3).

In the increased mesiobuccal access group (*n* = 10) the maxillary fourth premolars were treated with root canal therapy using a 4 mm mesiobuccal transcoronal access (twice the size of the access created in the control group), and a 2 mm distal root canal access (Figure 2B). Access into the pulp chambers was made with a #330 pear bur on a high-speed dental hand piece. The access was enlarged circumferentially, and diameter was measured in both a mesial to distal direction as well as coronal to apical direction with a periodontal probe confirming the access was 4 mm in diameter. Cleaning, filing, shaping, and restoration were performed in the same fashion as the control group.

In the direct palatal access group (*n* = 10) the maxillary fourth premolars were treated with root canal therapy using a mesiobuccal and distal access size identical to the control group with an additional 2 mm access directly over the palatal root cusp (Figure 2C). Access into the pulp chambers was made with a #330 pear bur on a high-speed dental hand piece. Cleaning, filing, shaping, and restoration were formed in the same fashion as the control group with the addition of a glass ionomer (Ionoseal, Voco, Cuhaven, Germany) and composite (Aelite Flo, Bisco, Anaheim, CA) restoration at the palatal root access site.

In the palatal hemisection and root extraction group (*n* = 10) the teeth were treated with root canal therapy using a mesiobuccal and distal access size identical to the control group; but with the addition of hemisection and complete extraction of the palatal root rather than obturation (Figure 2D). Hemisection of the palatal root was performed using a #699 crosscut, fissure bur, on a high-speed dental handpiece beginning at the furcation of the mesial roots. The hemisection site was restored using glass ionomer (Ionoseal, Voco, Cuhaven, Germany). Cleaning, filing, shaping, and restoration of the mesiobuccal and distal root access sites was performed in the same fashion as the control group.

### Biomechanical Testing

The root canal treated maxillary fourth premolar teeth were stored in containers of lactated ringer's solution at room temperature until the time of testing. Biomechanical testing was performed within 5 days of root canal treatment for all teeth. All specimens were potted into polytetrafluoroethylene rings filled with polymethyl-methacrylate (PMMA) (Ortho-Jet, Lang Dental, Wheeling, IL). The samples were placed into acrylic at a level just below the cementoenamel junction and placed as perpendicular as possible to the bottom surface of the ring. The teeth were kept moist with gauze saturated in lactated ringer's solution to allow the PMMA to cure for 1 h. After curing, the teeth potted in the PMMA were stored in lactated Ringer's solution at room temperature (20°C) until testing. Biomechanical testing was performed within 48 h of seating the teeth in acrylic.

**Figure 4:**
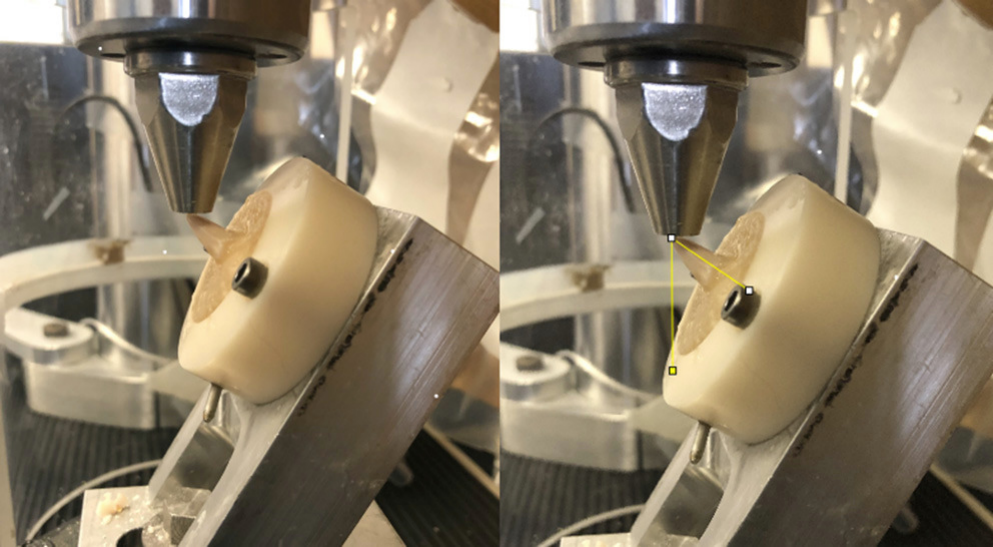
Biomechanical testing was performed in a universal testing machine with a 9 mm metal rod applying force over the palatal aspect of the main cusp with a 60° angle between the applied load relative to the long axis of the tooth. Angle between direction of applied load and long axis of the tooth was determined using image J (National institute of health, Bethesda, MD). The angle measured in this example was 55°.

Samples were randomized prior to testing. Loading was performed in a similar manner as previously described for determination of fracture limits of maxillary fourth premolar teeth (23). Briefly, the polytetrafluorethylene rings were securely mounted on a custom-built aluminum device designed to hold each cylinder at an angle of 60° with respect to the ground. This loading angle was chosen based on previous preliminary fracture testing of maxillary fourth premolar teeth (23), which showed that loading angles of 50–70° created fracture patterns most consistent with those clinically observed and defined by the American Veterinary Dental College (AVDC). Furthermore, previous computed tomography images using an object placed in between the maxillary fourth premolar and mandibular first molar has shown that although the impact angle is dependent on how far the object is inserted into the mouth, a 60° impact angle on the palatal aspect of the maxillary fourth premolar tooth is within the possible angles of impact during normal canine chewing (23). A 9 mm diameter stainless steel rod was used to apply the compressive force on the palatal aspect of the cusp tip (Figure 4). Photographs were taken of each sample prior to loading and the angle of impact was calculated using Image J (National institute of health, Bethesda, MD) (Figure 4). All samples were subjected to a 10 N preload for 10 s and then tested to fracture at a rate of 0.1 mm/s. The 10 s preload time period was eliminated from the force deflection curve for all samples. The force at fracture was classified as an instantaneous decrease of force on the force deflection curve >50%. Fracture type was categorized according to American Veterinary Dental College classification (25).

### Statistical Analysis

To explore differences in distal root canal volume between the treatment groups, a one-way analysis of variance (ANOVA) was performed. To explore associations between the maximum force (N) prior to fracture and treatment group, crown height to diameter ratio, distal root canal volume, and impact angle, both univariate models and multivariate models were fit. For categorical variables (group), estimated marginal means are reported, and for continuous variables, standardized coefficients (the average change in force per standard deviation increase) are reported. Pairwise differences were assessed using the Tukey adjustment for multiple comparisons. All models were checked for equal variance and normality of residuals using boxplots and qq-plots. Models were fit both with and without two potential outliers; for appropriate comparisons, the standardized coefficients for all models were computed using standard deviations from the entire data set. Additionally, to test for an association between type of fracture and treatment group, Fisher's test was used. For all results, *p*-values and 95% confidence intervals are reported, and significance was set at *p* < 0.05.

## Results

**Figure 5:**
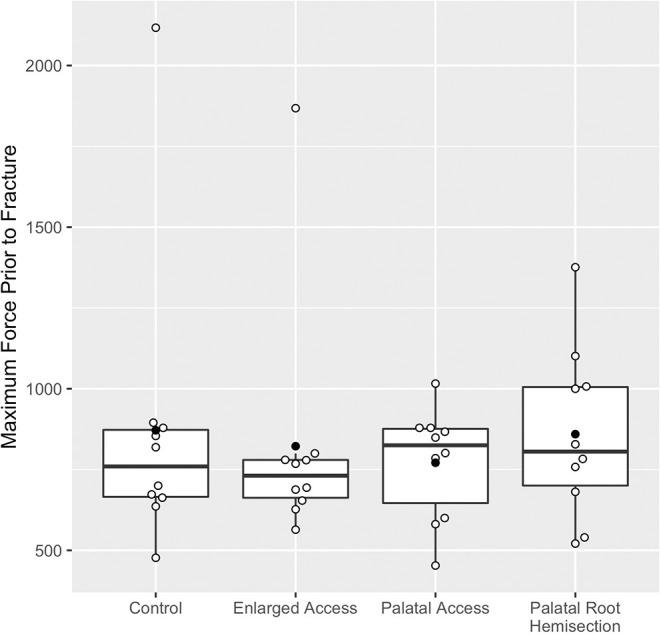
Boxplots of maximum force sustained prior to fracture for each treatment group. Data points shown in open circles and means for each group shown in closed circles. Please note the two outliers, one in the control group with a maximum force prior to fracture of 2,117 N and one in the enlarged access group with a maximum force prior to fracture of 1,868 N.

The mean (± standard deviation) of the maximum force sustained prior to fracture for all teeth was 831 N (± 324 N) at a mean impact angle of 57.3° (± 3.8°). The force sustained prior to fracture for the control and the alternative treatment groups is shown in Figure 5. There was no statistically significant difference in mean force to fracture between the treatment groups or between the control and treatment groups (*p* = 0.91). The largest observed difference was between the palatal access group (mean of 771 N; 95% CI: 556, 986) and the control group (mean of 871 N; 95% CI: 656, 1,086).

There were two potential outliers identified within this study, both of which fractured at forces more than twice the mean. The outliers included one tooth in the control group with a maximum force prior to fracture of 2,117 N, and one tooth in the enlarged mesiobuccal access group with a maximum force prior to fracture of 1,868 N. These teeth did not come from the same specimen, did not suffer the same fracture type, and were not significantly different from the remainder of the specimens in any other way.

When the outliers were excluded, estimates for force to fracture were notably more precise, though the differences were still not statistically significant (*p* = 0.28); the largest difference was then between the enlarged mesiobuccal access group (mean of 706 N; 95% CI: 584, 828) and the palatal hemisection and extraction group (mean of 860 N; 95% CI: 744, 975).

There was no overt difference in structural or loading variables between the treatment groups (Table 1). Differences in distal root canal volume between treatment groups were not statistically significant (*p* = 0.17); the largest observed difference was between the direct palatal access group (mean of 26.1 mm^2^) and the enlarged mesiobuccal access group (mean of 38.2 mm^2^). Furthermore, on evaluation of the force displacement curves, no overt differences in the slope of the curves to suggest differences in tooth stiffness were seen between the treatment groups.

**Table 1:** Mean and standard deviations (SD) of structural and loading variables by group.

**Treatment group**	**Mean crown height (mm)**	**Mean crown diameter (mm)**	**Mean height to diameter ratio**	**Mean distal root canal volume (mm^**2**^)**	**Mean impact angle (°)**	**Mean maximum force prior to fracture (*N*)**
Control group	10.58 (0.68)	15.47 (0.78)	0.68 (0.03)	30.8 (14.00)	57.1 (3.2)	871 (457)
Control group with outliers removed	10.54 (0.71)	15.50 (0.82)	0.68 (0.03)	32.4 (13.8)	57.2 (3.3)	733 (139)
Enlarged mesiobuccal access group	10.86 (0.67)	15.52 (0.82)	0.70 (0.05)	38.2 (14.5)	58.3 (1.9)	822 (375)
Enlarged mesiobuccal access group with outliers removed	10.76 (0.62)	15.31 (0.51)	0.70 (0.05)	38.9 (15.2)	58.5 (1.9)	706 (81)
Palatal access group	10.78 (0.62)	15.26 (0.57)	0.71 (0.04)	26.1 (8.1)	57.0 (4.3)	771 (172)
Palatal root hemisection group	10.83 (0.46)	15.92 (0.91)	0.68 (0.03)	29.7 (10.8)	56.9 (5.3)	860 (265)

On analysis of potential contributors to resulting force to fracture other than treatment group, there was not a statistically significant association between distal root canal volume and resulting maximum force to fracture (*p* = 0.16). Notably, a one standard deviation increase (12.5 mm^2^) in distal root canal volume was associated with a 73.2 N decrease in force to fracture (95% CI: −177, 30.6). When outliers were excluded from evaluation, the association between distal root canal volume and force to fracture was still not statistically significant (*p* = 0.25), but the estimated decrease per standard deviation increase is notably smaller and more precise, at 34.4 N (95% CI: −94.6, 25.8).

There was also no statistically significant correlation between the crown height to diameter ratio of each tooth and the maximum force sustained prior to fracture (*p* = 0.79). However, a one standard deviation increase in crown height to diameter ratio (0.04) was associated with a 14 N decrease in force to fracture (95% CI: −121, 93). When outliers were excluded, this association was still not statistically significant (*p* = 0.86), although the estimated decrease per standard deviation increase is again notably more precise, at 24 N (95% CI: −84, 36).

Lastly, there was no statistically significant association between impact angle and maximum force to fracture (*p* = 0.81). A one standard deviation increase in impact angle (3.8°) was associated with a 12.6 N increase in force to fracture (95% CI: −94, 119). When outliers were excluded, this association was still not statistically significant (*p* = 0.25), although the estimated increase per standard deviation increase is notably larger and more precise, at 34 N (95% CI: −25, 93).

As outlined above, there was no statistically significant difference when outliers were excluded, however, all estimates became notably more precise, so results from both analyses are included. All analyses were performed both as univariate analyses and as multivariate analyses with all four variables of interest (treatment group, distal root canal volume, crown height to diameter ratio, and impact angle) as predictors. The multivariate analyses gave similar results, so only the univariate analyses are reported here.

The most common fracture type that occurred was a complicated crown fracture (*n* = 26), occurring in 65% of teeth, followed by complicated crown root fracture (*n* = 7), which occurred in 17.5% of teeth. Root fractures were the third most common fracture type (*n* = 5), occurring in 12.5% of teeth. Overall, there was no significant difference in the prevalence of fracture types between the treatment groups or between the control and the treatment groups (Table 2).

**Table 2:** Frequency of fracture type by treatment group.

**Group**	**Uncomplicated crown fracture**	**Complicated crown fracture**	**Complicated crown root fracture**	**Root fracture**	**Total**
Control	1 (10%)	7 (70%)	2 (20%)	0 (0%)	10
Enlarged mesiobuccal access	0 (0%)	8 (80%)	1 (10%)	1 (10%)	10
Palatal access	0 (0%)	5 (50%)	3 (30%)	2 (20%)	10
Palatal root hemisection and extraction	1 (10%)	6 (60%)	1 (10%)	2 (20%)	10
**Total**	2 (5%)	26 (65%)	7 (17.5%)	5 (12.5%)	40

## Discussion

The present study is the first to report on fracture resistance of root canal treated maxillary fourth premolar teeth in dogs. It was found that the mean force to fracture in the endodontically treated teeth was decreased compared to structurally sound maxillary fourth premolar teeth loaded in a similar manner (23). Specifically, the mean maximum force to fracture of intact teeth was previously reported as 1,281 N (23), while in the present study the mean maximum force was 831 N, suggesting that endodontic therapy in dogs results in decreased fracture resistance similar to humans (7–13). Conversely, direct comparison of fracture resistance between structurally sound teeth in the previous study and endodontically treated teeth in the current study is difficult due to the inability to control for many important variables between study populations including crown height to diameter ratio, hard tissue volume, age, breed, weight, and dental history. Notably, there was a slightly higher average crown height to diameter ratio within this study population compared to the Soltero-Rivera study (23) (0.69 compared to 0.60), which may have directly contributed to the decreased fracture resistance, as this is a known confounder of tooth strength in dogs (23, 26, 27).

Although the present study did not find a statistically significant correlation between crown height to diameter ratio and fracture resistance within the population, our analysis estimates that for each standard deviation increase in crown to height diameter ratio (0.04) there was an associated decrease of 14 N (95% CI: −121, 93) in the resulting force to fracture. For reference, the difference between the crown height to diameter ratio in the Soltero-Rivera Paper (23) was 2.38 standard deviation lower than our mean, and so we would predict an average difference of 33 N (95%CI: −288, 221) in force to fracture between the two study cohorts based on differences in crown height to diameter ratio alone. Thus, our study is not in conflict with the findings of numerous other biomechanical studies (23, 26, 27) that found there is a trend for decreased fracture resistance as the height to diameter ratio increased.

Overall, the authors believe the lack of identified statistical significance was primarily due to the minimal variation in crown height to diameter ratio in the cohort, as was intentional in the study design, rather than the height to diameter ratio not being a significant contributor to overall fracture resistance in endodontically treated maxillary fourth premolar teeth. An additional consideration is the method of measurement for the crown height to diameter ratio. In the present study the crown diameter was measured from the most mesial to most distal aspect of the crown, as previously described (23, 26). However, alternative methods of crown diameter measurement, such as measurement in a buccal to palatal direction may have been more appropriate, and thus more closely associated with fracture strength given the palatal to buccal load direction that was tested.

The authors further caution against direct comparison of these biomechanical studies due to variation in biomechanical testing including use of a different universal testing machine and variation in aluminum jig design resulting in differences in loading angle and direction of force applied relative to the cusp of the tooth. These variables resulted in differences in total surface area where the load was placed and distributed, and thus likely resulted in differences in resulting maximum force to fracture. Additionally, the loading angle was evaluated in a different method than previously described (23). In the present study, the angle between the direction of force relative to the long axis of the tooth was calculated, whereas in a previous study the angle between the face of the actuator relative to the long axis of the tooth was evaluated (23). This also resulted in ultimate differences in exact angle of impact and likely affected force to fracture. For more accurate comparison, evaluation of untreated maxillary fourth premolar teeth as a control was considered, but due to limited cadaveric samples this was ultimately not possible without further limiting the sample size per group and the ability for relevant statistical analysis.

Thus, although this study suggests that endodontic therapy may weaken the overall strength of the tooth, further veterinary studies are needed to confirm the effect of root canal therapy on fracture resistance. As endodontic treatment in veterinary patients is generally only indicated in teeth that have undergone previous trauma, it would also be important to consider whether changes in fracture resistance are primarily due to previous trauma sustained by the tooth or due to the biomechanical changes that are directly related to endodontic therapy. Specific areas of interest would be evaluation of the effect of cyclic loading and tooth abfraction (28–30) on fracture resistance compared to the direct effects of root canal therapy, such as dentin desiccation and specific access design (14–20).

In humans, where the effect of endodontic treatment on tooth strength has been clearly defined, the access method has been identified as one of the most important variables affecting fracture resistance post therapy (14–19). This is directly related to the fact that more conservative access methods preserve coronal hard tissue, namely dentin, resulting in a higher ultimate strength of the tooth (14–19). Surprisingly, the present study did not confirm this finding to be the same in dogs, and no significant difference was found between the fracture resistance of the control group with a small mesiobuccal endodontic access and a treatment group with a mesiobuccal access twice as large in diameter nor between the control group and the treatment group with an additional access site.

Despite the lack of significance, it is important to note that there was a trend for the alternative treatment groups with enlarged or additional access sites to have a decreased force to fracture compared to the control. Specifically, when the outliers were included in analysis the additional palatal access group had the lowest force to fracture (mean of 771 N; 95% CI: 556, 986), and with the outlier excluded the enlarged mesiobuccal access sites had the lowest force to fracture (mean of 706 N; 95% CI: 584, 828).

Human studies focused on the effect of endodontic access cavity preparation compare specific access designs such as traditional endodontic cavity vs. conservative endodontic cavity rather than strict access size in millimeters (mm). This is because posterior teeth that require root canal therapy generally have a history of extensive carious lesions and previous restoration. Thus, there is no specific recommendation in ideal access size, as removal of previous restorations or carious lesions dictates the size of the access (10). Micro-CT is then most often utilized in studies to identify the mean volume of dentin removed for different access cavity preparations to evaluate the overall differences in amount of tooth structure removed on resulting fracture resistance (18).

Given the lack of an exact access size defined in the human literature to model this canine biomechanical study on, a clinically relevant “conservative access size” was chosen for the control group, and the alternative testing groups were then modeled based on this access. It is plausible that doubling the access size was not a large enough change in overall tooth structure to truly define the effect of access design on fracture resistance in canine teeth. To better control and evaluate for hard tissue loss, additional studies in veterinary endodontic therapy could benefit from micro-CT to more closely evaluate the amount of tooth structure removed with different access methods and define at what point long-term tooth strength is significantly affected.

The fact that endodontic access method was not shown to affect fracture resistance may also be in part due to the fact that there are significant anatomical and functional differences in human and canid teeth, including different tooth morphology, chewing forces, masticatory patterns, and enamel thickness that would all be expected to contribute to differences in fracture risk and long-term fracture resistance. Not only do humans have primarily bunodont dentition with low rounded cusps primarily made for grinding, but they also have much thicker enamel in the range of 2–4 mm (2). Comparatively, the canine maxillary fourth premolar is a secodont tooth specifically designed for shearing with an enamel thickness ranging from 0.1 to 0.6 mm (2). Therefore, while in human teeth the loss of additional dentin and enamel secondary to larger access sizes may contribute significantly to decreased fracture resistance against normal occlusal forces, this may not be true for canine maxillary fourth premolar teeth that generally experience brief periods of heavy shearing forces during chewing of toys or bones. Finally, it is also difficult to directly compare the biomechanical testing studies performed in humans and canines. The biomechanical testing method used in this study was fast fracture or overload fracture secondary to application of compressive force only, whereas in many human studies fracture resistance is evaluated using either slower compressive forces or cyclic fatigue fracture methods to better mimic normal occlusal forces sustained by human molars. These anatomic, functional, and biomechanical testing differences may be an explanation for the finding that access size does not appear to have a significant effect on fracture resistance of root canal treated maxillary fourth premolar teeth in dogs, which is in direct contrast to the findings in human dentistry. Furthermore, it is important to note that although the mean force to fracture was not statistically significant between treatment groups, the average differences in fracture resistance between the control group and treatment groups or between the different treatment groups may be as large as 504 N, which actually may represent a clinically impactful difference. Clinical *in vivo* studies evaluating the long- term outcome following alternative palatal root treatment options would help determine if there truly is no difference in long term fracture resistance with enlarged or additional access sites for difficult to instrument palatal roots.

The present study also evaluated the effect on maximum force to fracture for teeth treated with hemisection and extraction of the palatal root. This has previously been discussed as a salvage option for endodontically inoperable roots that cannot be properly sterilized and obturated (2). This technique was anticipated to significantly alter the biomechanics of the tooth as it creates a point of increased stress distribution at the cemento-enamel-junction of the remaining buccal roots. However, no statistically significant difference was found between the fracture resistance of the hemisection group and the control, nor with any of the other treatment groups. In fact, palatal root hemisection had the highest mean force to fracture of all the groups.

These findings suggest that palatal root hemisection and extraction can be viewed as an acceptable treatment option for the palatal root with minimal effects on overall strength of the tooth. *In vivo* studies, however, are required to confirm this finding as numerous differences may exist in a clinical situation; the primary being the effect of the surrounding alveolar bone on the biomechanics of tooth fracture. Clinically, the palatal root alveolus would gradually fill with bone (31). In the present study, the alveolus was allowed to fill with PMMA, which is unlikely to properly mimic the biomechanics of woven bone. Additionally, the present study only evaluated a single tooth with its surrounding alveolar bone, which does not account for the true biomechanics of a single tooth as part of a complete dentition and associated supporting bone of the entire maxillary arch.

It is also possible that forces applied in a different direction, such as buccal to palatal would have resulted in significant differences in fracture pattern (i.e., increased root fractures) for this treatment group. However, in the authors' opinion, the selection of force from a palatal to labial direction is the most clinically relevant application of force due to the relationship between the mandibular first molar tooth and maxillary fourth premolar tooth and is consistent with loading direction from other canine biomechanical fracture studies (23).

No treatment methodology increased susceptibility to a specific fracture type. The two most common types of fracture that occurred were complicated crown fractures and complicated crown root fractures, occurring in 65% of teeth and 17.5% of teeth, respectively. This is consistent with the clinical incidence of premolar fracture types, with enamel-dentin-pulp fracture and crown root fracture with pulp exposure being the most common types of fracture in premolar teeth (1, 23).

One surprising finding was the frequency of root fractures in this study which occurred in 12.5% (*n* = 5) of teeth tested. Previous epidemiological studies of dentoalveolar injury have shown root fracture to be less likely in strategic teeth (1). As there was no significant difference between type of fracture and endodontic access method, the frequency of root fracture in this study may be secondary to the alternative factors, such as the direction of the applied force (palatal to labial) used during biomechanical testing or changes to biomechanics from testing a single tooth potted in PMMA compared to an entire maxillary arch. Additionally, a study evaluating fracture resistance of endodontically treated teeth in humans has shown that radicular dentin is weaker after endodontic therapy, leaving the tooth at increased risk for vertical root fracture (20), thus it is possible changes to dentinal strength may also have contributed to this finding.

Although there was no statistically significant difference between type of fracture and access method, no root fractures occurred within the control group, while at least 1 root fracture was seen in all treatment groups. All root fractures seen in this study were cervical horizontal root fractures. In the enlarged mesiobuccal access group the prevalence of root fractures was 10% (1/10) and in both the palatal access and palatal root hemisection groups the prevalence of root fractures was 20% (2/10). Thus, when the palatal root segment was treated, the frequency of root fracture doubled. This finding suggests that treatment of the palatal root through either hemisection or direct access may increase the susceptibility of the tooth to cervical root fracture. However, this needs to be interpreted with caution due to the limited sample size and limited overall number of root fractures. Additional studies are necessary to further evaluate whether access method of the palatal root segment affects root fracture susceptibility.

The authors acknowledge limitations of the present study, most notably, the small sample size and *in-vitro* nature of the study. Due to limited availability of cadaver teeth with similar pulp cavity sizes, the group size was relatively small and may have led to underpowered results during statistical testing. Ideally, untreated maxillary fourth premolar teeth would also have been biomechanically tested, but this was not performed due to the limited availability of similarly matched cadaveric maxillary fourth premolar teeth. Given the *in-vitro* nature of this study, the biomechanics of the periodontal ligament space, dentin/enamel biomechanics, and alveolar bone may not have been properly represented due to sample processing and storage (i.e., freezing). However, previous studies have found no detectable effect of freezing, or storage temperature in general, on the behavior of human periodontal ligaments or fracture resistance when tooth strength is evaluated by a force displacement curve (32, 33). Furthermore, the storage and harvesting methods used in this study were consistent with previously reported methods in canines (22, 26, 27). Based on this information, the authors believe it was unlikely that storage conditions had a significant effect on the results of the present study, as all teeth were stored and tested in the same manner. To the authors' knowledge ideal storage conditions and potential effects of storage conditions and time to biomechanical testing have not yet been determined for dental biomechanical testing in dogs.

In conclusion, given the limitations of an *in-vitro* study, the results of this study do not support the hypothesis that an increased access size will result in decreased fracture resistance of endodontically treated maxillary fourth premolar teeth. No treatment group was found to have a statistically different mean fracture resistance or resulting fracture pattern. Although the findings lacked statistical significance, it should be noted that the average differences in fracture resistance between the control group and treatment groups or between the different treatment groups themselves may be as large as 504 N. Thus, at this time we do not have enough evidence to provide guidance on which treatment method has the least effect on overall tooth strength. Therefore, when a clinician is faced with a difficult to instrument palatal root during endodontic treatment of a maxillary fourth premolar treatment the alternative access method selected can be based on operator experience, preference, and any specific anatomic variations affecting access such as size of the pulp cavity, pulp stones, or iatrogenic endodontic blockage.

## Data Availability Statement

The raw data supporting the conclusions of this article will be made available by the authors, without undue reservation.

## Ethics Statement

Ethical review and approval was not required for the animal study due to the cadaveric nature of the study.

## Author Contributions

JM: development and design of the study, sample harvesting, endodontic treatment, biomechanical testing, interpretation of the data, and drafting of the manuscript. SG: development and design of the study, interpretation of the data, revising the manuscript, and final approval of the version to be published. AR: statistical analysis and interpretation of the data. All authors contributed to the article and approved the submitted version.

## Conflict of Interest

The authors declare that the research was conducted in the absence of any commercial or financial relationships that could be construed as a potential conflict of interest.
